# Possible Biological Mechanisms Linking Mental Health and Heat—A Contemplative Review

**DOI:** 10.3390/ijerph15071515

**Published:** 2018-07-18

**Authors:** Mare Lõhmus

**Affiliations:** 1 Centre for Occupational and Environmental Medicine, Stockholm County Council, Solnavägen 4, 113 65 Stockholm, Sweden; mare.lohmus.sundstrom@ki.se; 2 Institute of Environmental Medicine, Karolinska Institutet, Nobels väg 13, 17177 Solna, Sweden

**Keywords:** heatwave, mental health, thermoregulation, neuroimaging, sleep

## Abstract

This review provides examples of possible biological mechanisms that could, at least partly, explain the existing epidemiological evidence of heatwave-related exacerbation of mental disease morbidity. The author reviews the complicated central processes involved in the challenge of maintaining a stable body temperature in hot environments, and the maladaptive effects of certain psychiatric medicines on thermoregulation. In addition, the author discusses some alternative mechanisms, such as interrupted functional brain connectivity and the effect of disrupted sleep, which may further increase the vulnerability of mental health patients during heatwaves.

## 1. Introduction and Aim

There is strong evidence that changing weather patterns associated with climate change are shifting the geographic range, seasonality, and transmission of certain diseases, and increasing morbidity and mortality associated with extreme weather events [1]. Due to the undertaken climate research, the understanding of the characteristics, timing, and spatial patterns of climate change has increased; and together with demonstrable changes in disease incidence and mortality, it has been possible to develop scientific methods that allow us to attribute a portion of the disease burden to climate change [1].

However, despite considerably increased interdisciplinarity in the research dedicated to climate change and human health associations, large gaps still exist that could be overcome by better collaboration between specialists in sometimes quite closely related branches of science. An increased exchange of information between neuroscientists working with the direct effects of heat exposure on human mental functions and the epidemiologist working with associations between ambient temperatures and mental disease morbidity/mortality would increase our understanding of this health burden. This review provides examples of possible biological mechanisms that could, at least partly, explain the epidemiological evidence of heatwave-related exacerbation of mental disease morbidity. Some areas of the topic, such as the maladaptive effects of certain psychiatric medicines during heatwaves and the decreased capacity for behavioral adaptation in mental disease patients, have been previously discussed in the literature, and are reviewed here only briefly. Some alternative mechanisms, such as interrupted functional brain connectivity and the effect of disrupted sleep, are discussed in more detail. The review focuses only on acute effects and does not cover secondary effects of heat waves on human mental health, such as the stress caused by loss of livelihood or bereavement. Articles cited in this paper were mainly found by multiple advanced searches on Web of Science (v.5.29) between October 2017 and July 2018, including titles or abstracts containing “heatwave”, “heat”, “temperature”, “hyperthermia” in combination with “mental health”, “mental illness”, “thermoregulation”, “central thermoregulation”, “neurotransmitter”, “drug”, “treatment”, “antidepressant”, “neuroleptic”, “antipsychotic”, “neuroimaging”, “brain connectivity” and “sleep”, as well as from references provided in the literature.

## 2. Climate Change, Heatwaves and Public Health

Climate change predictions indicate warmer temperatures and higher frequencies of extreme events [2]. Globally, extreme temperatures are among the 10 worst reported natural disasters in terms of lives lost since the 1970s [3]. In Europe, heat-related mortality, during the same period, was higher than any other kind of disaster-related mortality [2]. Vulnerability to heatwaves varies on a regional level due to different climatic conditions, population characteristics, and local adaptation measures. However, individual heat vulnerability depends on a combination of both person-specific (age, gender, income, and ethnicity) and contextual (population density, urban characteristics, socio-economy, and access to health services) factors, and may differ not only between countries or municipalities, but also between closely situated neighborhoods in the same city [2,4].

On a population level, the groups of citizens with increased risk of heat-related illness include the elderly and people with chronic physiological (such as cardiovascular and respiratory disease, long-standing diabetes and obesity) and mental health conditions [5]. Physiological health conditions often impair the body’s ability to regulate core temperature in hot environments. However, in many cases, the medications that are used to treat health conditions–both physiological and mental–contribute to a reduced thermoregulatory capacity in the heat. A homebound lifestyle and lack of contact with other people has been shown to further increase the vulnerability, and similar to other health risks, heat stress is more likely to affect residents at the lower end of socio-economic spectrum [6].

## 3. Associations between Heatwaves and Mental Disease Exacerbation

The interest in detecting and preventing heatwave-related morbidity/mortality increased considerably after the 2003 European heatwave that was suggested to have caused more than 70,000 additional deaths in total in 16 European countries [7]. After this catastrophic event, both the scientific community and regional governments and institutions in many places increased their efforts to quantify the health effects of, and increase the local resilience to, heatwaves. Many studies were published about the vulnerability analyses of specific groups in society. One of these groups, where the evidence of heat vulnerability was often found to be stronger than in any others, was the population suffering from different kinds of mental or behavioral disorders [8].

The knowledge that ambient temperature causes psychotic exacerbation of core symptoms in sufferers of mental and behavioral disorders has existed and drawn attention for many years prior to 2003. For example, during the 1970s, the New York State psychiatric hospital experienced a large number of deaths among patients during heatwaves. To reduce this heat-related mortality, major preventive measures, such as air-conditioning and close monitoring of the patients, were instituted in the beginning of 1979 [9]. Bark [9], who analysed the mortality data in the New York State psychiatric hospital from 1950 to 1984, stated that during this period the psychiatric patients had double the risk of dying during a heat wave as the general population. Bark also observed that the risk of dying was higher in the 1950s, before the large-scale introduction of the antipsychotic medications, than in the 1980s. However, the mortality was even higher in 1970s when very high doses of certain medications were used. The author concluded that both psychiatric illness and the usage of antipsychotic medication contributed to the increased risk of dying during heatwaves.

In 1996, Semenza et al. [10] showed increased odds ratios (OR) for death during the California heatwave in 1995, among others, in persons with pre-existing mental health conditions (OR 3.5, 95% confidence interval (CI) 1.7–7.3). The odds ratio for this group was higher than for groups with any other pre-existing chronic diseases, such as cardiovascular and pulmonary conditions (OR 2.3, 95% Cl, 1.5–3.6 and 2.2, 95% Cl, 1.0–4.9, respectively). In a second paper, Semenza et al. [11] observed a 20% increase in hospital admission in patients with Central Nervous System (CNS) disorders during the same period. Mental disorders were also a significant contributor to the 2003 heatwave mortality in Paris (748+ deaths); however, their impact was found to be lower than that of circulatory system (3004+ deaths) and respiratory (1365+ deaths) causes [12]. Another study analysing the Paris 2003 heat wave effects on mortality, specifically among the community-dwelling elderly, demonstrated that mental disorders had a stronger effect on excess mortality in this population group than any other medical precondition (OR 5.86, 95% Cl 2.75–12.49) [13]. In a meta-analysis performed by Bouchama et al. in 2007, the authors found that, among preexisting conditions psychiatric illness tripled the mortality risk and was the factor that was most strongly associated with death during heatwaves (OR 3.61, 95% Cl 1.3–9.8). Other conditions significantly contributing to mortality included: cardiovascular illness (OR 2.48, 95% Cl 1.3–4.8), taking psychotropic medications (OR 1.90; 95% CI 1.3–2.8), and pulmonary diseases (OR 1.61; 95% CI, 1.2–2.1), among others [8].

After 2003, an increasing number of epidemiological studies specifically focusing on temperature effects on mental health were performed. In 2008, Hansen et al. [14] estimated the effect of heat waves on hospital admissions and mortalities attributed to mental, behavioral, and cognitive disorders in Adelaide, South Australia, for 1993–2006. They reported a 7.3% increase in hospital admissions above an ambient temperature threshold of 26.7 °C for all mental diseases. Elevated air temperatures had a significant effect on hospital admission rates due to organic illnesses; dementia; mood affective disorders; neurotic, stress related, and somatoform disorders; disorders of psychological development; and senility [14]. Furthermore, the authors observed an increase in mortalities attributed to mental disorders in the 65- to 74-year-old age group, and in schizophrenia, schizotypal, and delusional disorder patients. Similarly, in Toronto, Canada, an association between higher temperatures and emergency room visits associated with mental and behavioral diseases with significant trends for schizophrenia, mood, and neurotic disorders, was reported [15]. Other studies, associating ambient temperatures and warm seasons to psychiatric emergencies and hospital admissions caused by mental disorders, included studies focusing on schizophrenia [16,17], depression [18], dementia [19,20], and mania [21], as well as on Parkinson’s [22] and Alzheimer’s diseases [23] and were conducted in Italy [19,24], Spain [20,22,23], Taiwan [16], China [17], Vietnam [18,25,26] and Denmark [21], among others.

## 4. Thermoregulation

In healthy people, the body attempts to maintain a core temperature at near-constant level at about 37 °C, irrespective of environmental temperature [5]. Cutaneous vasodilation, which increases the skin blood flow in a warm environment, is an adaptive response that brings the metabolic heat to the body surface and thereby facilitates the transfer of heat to the surrounding environment [27]. In addition, evaporation of sweat from skin surface is important for heat loss. The sweat response, however, is dependent on cutaneous vasodilation, as it provides blood plasma as the necessary precursor fluid for sweat production [28]. In humans, increased blood flow in the skin is accompanied by a vasocontraction (decreased blood flow) in visceral organs [27]. To compensate for the decreased blood flow in visceral areas, an increase in cardiac output, allowing sufficient delivery of oxygen to inner organs, is necessary [5].

### 4.1. Thermoregulatory Challenges in Body and Brain

Under circumstances in which changes in sweating and skin blood flow do not lead to sufficient heat loss, body core temperature increases continuously, leading to a condition called hyperthermia. Heat stroke, the most severe complication of heat exposure, is a life-threatening condition characterized by a core temperature higher than 40 °C and dysfunctions in most organs, including the central nervous system. Prior to the development of heat stroke, however, signs including fatigue, weakness, dizziness, and decreased consciousness are observed [29]. Prolonged exposure to heat strain can increase the likelihood of heat-related illness.

Exposure to heat also affects the temperature and function of the brain. Heat is produced by the brain itself when it consumes oxygen, and is removed by blood flow [30]. Accordingly, increased blood flow is the stabilizing, adaptive response to increased brain temperature. However, changes in the temperature of incoming blood might affect the efficiency of the brain cooling process [30]. Since temperature affects the affinity of hemoglobin for oxygen, changes in blood temperature also change the blood oxygen saturation levels [30]. Consequently, increasing body temperatures might influence oxygen delivery to different brain regions and thereby influence the information processing procedure of the CNS.

Among the pathophysiological effects of hyperthermia, increased permeability of the blood-brain barrier (BBB) has been reported [31]. The BBB is a highly selective semipermeable membrane barrier that separates the circulating blood from the brain and extracellular fluid in the CNS. The ultra-selective permeability of BBB is evolutionarily adaptive, as it makes it difficult for pathological organisms to attack CNS. Increased BBB permeability in hyperthermia may therefore increase the vulnerability of the brain to both pathogen organisms and toxic neurochemicals [31]. The pathology of severe hyperthermia includes edemas and swollen nerve cells with disintegrated nuclei in various brain areas and exerts often irreversible neurological damage [31].

### 4.2. Central Thermoregulation

The maintenance of a stable body temperature is a complex process that involves a myriad of interrelated functions. Here, we briefly summarize the present understanding of the peripheral and central thermoregulatory networks in warm environments, as some aspects are important for understanding the later discussion about vulnerability of mental health patients during heat waves. For more detailed reviews, please read [27,28,32,33].

Environmental temperature, both cold and warm, is primarily sensed by peripheral cutaneous thermoreceptors. The molecular mechanisms mediating this thermal sensation involve the transient receptor potential (TRP) family of cation channels [27]. TRPV3 and TRPV4 are the warm-sensitive TRP channels activated by high ambient temperatures. When epithelial cells (keratinocytes) expressing TRPV3 and TRPV4 ion channels are stimulated by elevated temperatures, they release signal substances, such as the cytokine interleukin-1 alpha, to adjacent sensory nerve endings [27]. The somatosensory nerve fibers then deliver the thermal signal to specific neurons in the dorsal horn of the spinal cord. Lamina I neurons are the best-known ascending thermosensory pathway conducting information from the spinal dorsal horn to the CNS. These neurons synapse directly in the thalamus, which then projects to the primary somatosensory cortex, allowing sensory perception of cutaneous temperature (i.e., the conscious feeling of “warm”). However, to activate the physiological thermoregulative processes in the body, the signaling pathway from lamina I neurons via lateral parabranchial nucleus to the preoptic anterior hypothalamus (POAH) has been suggested to be of greater importance [27,32]. In addition to cutaneous thermoreception, thermoreceptive mechanisms, including the TRP channels, are also present in body core structures, such as the brain, spinal cord, and visceral organs [27]. The principal thermoreceptive mechanisms at these locations are similar to those of the cutaneous system. However, afferent nerve fibers from warm receptors in viscera are suggested to conduct sensory signals to the CNS via the splanchnic and vagus nerves, whereas the somatosensory fibers in the spinal dorsal horn mediate temperature sensation in the spinal cord. The POAH also contains abundant neurons that sense changes in local brain temperature [27].

While the anatomical physiology of thermoregulation is understood, the central neuro-molecular regulation of it is not as well known. Neurotransmitters such as serotonin, acetylcholine, norepinephrine, dopamine, prostaglandin, gamma-aminobutyric acid (GABA) and adrenocortopic hormone are all involved, but the precise pathways for individual neurotransmitters are not clear. The POAH is often characterized as the primary central structure of thermoregulation that receives, integrates and weighs both the central and peripheral signals. Temperature sensation in POAH is thought to occur through a population of GABAergic neurons (neurons where GABA acts as the neurotransmitter) with intrinsic warm sensitivity [28,32]. In addition to the POAH, other brain centers, such as the perifornical lateral hypothalamus (PeF/LH), the paraventricular nucleus (PVN), and the ventral tegmental area (VTA), are reported to be involved in thermoregulation [32].

Triggered by increased core and skin temperatures, impulses from the POAH and other brain areas lead to the activation of cutaneous cholinergic (neurons where acetylcholine acts as the neurotransmitter) sympathetic nerves carrying signals to both sweat (eccrine) glands and to blood vessels [34]. These signals allow adaptive physiological responses, such as cutaneous vasodilation and thermoregulatory sweating. In thermoregulatory sweating, in addition to cholinergic neurons, noradrenergic neurons are thought to play an important regulatory role. Common signaling molecules for the vasodilatory and sudomotor (sweating) responses include calcitonin gene-related peptide, substance P, vasoactive intestinal polypeptide, and acetylcholine. These systems are complex and poorly understood, and additional presently unknown mechanisms may trigger specific physiologic responses [28].

As the production of sweat requires water, adaptive thermoregulation is dependent on adequate hydration. Thirst is the body’s defense mechanism in response to central perception of a deficit in body fluids. Key physiological signals for thirst include increased plasma osmolality and consequential cellular dehydration and hypovolemia (a decrease in the blood plasma volume). The osmoreceptor cells are mainly located in a forebrain structure called the lamina terminalis. These receptors activate the central system for antidiuretic hormone (ADH, also called vasopressin) secretion [35,36]. The release of ADH is mediated by peripheral baroreceptors, cardiopulmonary volume receptors, and the hormone angiotensin II, and results in decreased renal water exertion and thirst stimulation [36].

In addition to POAH, the involvement of the PVN in thermoregulatory functions is of considerable importance. The thermoregulatory signaling pathways between POAH and PVN are not clear but have been suggested to involve GABAergic (inhibitory) and glutamatergic (excitatory) inputs from the POAH [37]. The PVN serves as the brain’s neuroendocrine control center that regulates the release of numerous circulatory hormones, including ADH, in the hypothalamo-pituitary-adrenocortical (HPA) axis. The thermoregulatory communication between POAH and PVN is evident from several functions, not least from the myriad of biological stress responses following persistent heat exposure [38]. Heat stress has been shown to increase the secretion of HPA hormones adrenaline, noradrenaline, and cortisol, but also increase the circulatory levels of serotonin and hypothalamic levels of both dopamine and serotonin [38,39], changes of which are not only likely to affect physiological responses, but affect mood state and cognition [39].

## 5. Behavioral State Neurotransmitters

Dopamine, serotonin, and, to some degree, noradrenaline, are probably the most notorious neurotransmitters. The general recognition of these monoamines is associated with their involvement in mood, memory, motivation, attention, intrinsic reward system, drug addiction, and in several mental and neurodegenerative diseases, which has led to some interest in the popular media [40,41,42]. The biological roles of these substances are, however, much wider and extremely complex.

### 5.1. Thermoregulation and the “Behavioral State Neurotransmitters”

Studies from several decades back suggested that serotonin, dopamine, and noradrenaline are involved in thermoregulatory processes. As antidepressants and antipsychotic medicines directly affect the levels and regulation of these monoamines, this involvement is of interest when discussing the maladaptive effects of antipsychotic medications during heatwaves i.e., [43]. However, since the specific roles and functional pathways of these neurotransmitters in thermoregulatory processes are, again, largely unknown, the estimation of the risks is complicated.

In 1963, Feldberg and Myers [44] stated that noradrenaline and serotonin are the two principal neurotransmitters in the hypothalamus that functionally mediate heat loss and heat gain mechanisms, respectively. Several decades later, the reality appears to be much more confusing. The experimental history after Feldberg and Myers’ study has produced rather contradictory and difficult-to-interpret data concerning the role of noradrenaline, serotonin, and dopamine systems in thermoregulation. These contradictions may have been partly caused by the methodological bi-effects of early experimental studies, but are most likely also due to the non-uniform effects of these neurotransmitters in different brain areas [45,46]. Furthermore, since many of the studies investigated these systems in exercise-induced hyperthermia, the results may not have always automatically translated to the physiological regulation of passive, ambient temperature-related hyperthermia. In addition, almost all of the experimental studies have been conducted either in vitro or in experimental animals, which raises some concerns about the applicability of the results to humans, especially as differences in thermoregulatory processes between different mammalian species have been observed [27].

The main sources of serotonin innervation are located in the dorsal raphe nuclei and median raphe nucleus in the forebrain [45]. These nuclei send serotonergic nerve fibers to many other brain areas, such as the olfactory bulb, hypothalamus, thalamus, hippocampus, amygdala, and cerebral cortex [45]. Since the 1960s, several studies have attempted to clarify the role of serotonin signaling in the POAH for body temperature with contradictory results i.e., [44,47]. In 2004, Ishiwata et al. concluded that POAH may not be the main location for thermoregulatory signaling of serotonin, as external infusion of serotonin in the POAH did not affect body temperature, nor did heat exposure change the serotonin release in this brain area in their experiment [48]. Based on other reports suggesting that the ventral tegmental area (VTA) of the midbrain is strongly involved in the regulation of thermoregulatory vasodilation [49,50], Ishiwata et al. [45] focused on studying the serotonin responses in this area. They found an extracellular increase in serotonin levels, but not in noradrenaline or dopamine levels, in the VTA during exposure to heat and concluded that the VTA is the key action area for serotonin in thermoregulation.

The largest sources of dopamine in the vertebrate brain are the substantia nigra and VTA. Axons of dopaminergic neurons project to many brain areas, such as the hippocampus, nucleus accumbens, frontal cortex, and striate nucleus (for detailed review, see i.e., [51]). Similar to serotonin, the history of dopamine research concerning the thermoregulatory actions of this hormone has been contradictory [46,52,53,54,55]. Today, dopamine is generally believed to improve heat tolerance during exercise and affect the dissipation of exercise-induced heat and metabolic rate recovery during the post-exercise period [43,56].

Neurons in the locus coeruleus, located within the pons of the brain stem, are the central source of noradrenaline. The axons of neurons in the locus coeruleus project to both sides of the brain and innervate tissue in wide-ranging areas, such as the brain stem, spinal cord, cerebellum, hypothalamus, thalamic relay nuclei, amygdala, and neocortex [57]. Noradrenaline has many physiological and neurochemical roles and can, in different contexts, be characterized as a neurotransmitter, neurohormone, or neuromodulator. Local application of noradrenaline in the POAH was suggested to increase body temperature [58] and to inhibit the activities of the warm-responsive neurons [59] leading to conclusions that noradrenaline is involved in central heat production mechanisms [60]. Quan et al. [61,62], on the other hand, reported that noradrenaline, microdialyzed to POAH, evokes a fall in core temperature, mediated by a reduction in metabolic rate. With certainty, noradrenaline is an important neurotransmitter involved in the central neurological mechanisms allowing thermoregulatory sweating.

The involvement of serotonin, dopamine, and noradrenaline systems in both thermoregulation and behavioral and mental states has led some researchers to assert that these monoamines also form a direct link between the exacerbation of mental disorders and thermoregulatory responses during heatwaves. However, since the precise pathways and roles of these monoamines and their receptors in the regulation of body temperature homeostasis and in mental disorders are unknown, drawing any such conclusions is difficult. On the other hand, when discussing the drug-induced vulnerability of mental health patients during heatwaves, the thermoregulatory characteristics of these neurotransmitters may be of high relevance.

### 5.2. Vulnerability of Mental Health Patients–Behavioral Changes and Medicines

Compared to the general population, mental health patients often experience poorer overall health and show increased morbidity and mortality in general. Mental illness has also been identified as a serious risk factor for death and illness during heat waves [8]. One of the underlying reasons may be that environmental awareness and the ability to initiate adaptive behaviors, such as increased fluid intake, appropriate planning of daily activities, or wearing appropriate clothing, may be compromised in some groups of mental health patients, such as those with Alzheimer’s disease, dementia, psychosis, schizophrenia, and developmental disabilities [14]. Accordingly, “Unable to care for oneself” has been identified as an important risk factor in heat-related mortality and may contribute to the ill-health in the above-mentioned groups of patients [8]. Another factor also contributing to the risk of death during heatwaves is the use of psychotropic medications. By interfering with physiological homeostasis, these medications increase the vulnerability in mental health patients, even when their cognition and ability to care for themselves is unaffected by the disorder [8]. Many antipsychotic, anticholinergic, antidepressant, sedative, mood stabilizing, and nervous system medicines increase heat vulnerability through inhibition of the adaptive thermoregulative activities of the body [63]. Some examples are given below.

Anticholinergic drugs are not specific for the treatment of mental health diseases. Nonetheless, some of them are used, for example, in the treatment of Parkinson disease as well as for mitigation of side effects caused by antipsychotic drugs [64,65]. The action of an anticholinergic drug involves the inhibition of parasympathetic nerve impulses by selectively blocking the binding of the neurotransmitter acetylcholine to its receptor. Acetylcholine functions as the neurotransmitter at both cholinergic sympathetic nerves carrying signals to sweat glands, and at muscarinic receptors in the sweat glands. Consequently, the anticholinergic drugs or drugs with anticholinergic effects impair sweating, reduce heat elimination, thereby increasing the vulnerability of their users in heatwaves [65].

Sympathomimetic drugs, especially those that act as agonists at the adrenergic α receptor, cause hyperthermia through increased cutaneous vasoconstriction (i.e., decreased skin blood flow) [66]. Some sympathomimetics also increase metabolic heat production by agitation-related expanded muscular activity [67]. The consumption of sympathomimetic drugs is not specific to mental health patients, even though some of the medications, such as amphetamines and ephedrine, have been used to treat symptoms of hyperactivity and narcolepsy, respectively [66]. Behavioral disorders, such as abuse of the illegal drug ecstasy or MDMA (3,4-methylenedioxy-methamphetamine), can lead to severe hyperthermia [66].

Neuroleptics (antipsychotics), such as phenothiazines, have both anticholinergic and central thermoregulatory effects [67] and their use is associated with increased risk of heat stroke [68]. The clinical antipsychotic effects of neuroleptics are a result of central dopamine blockade. Unfortunately, the same mechanism also seems to affect central thermoregulatory capacity. The blockade of dopamine receptors in the hypothalamus is believed to result in impaired heat dissipation via impaired vasodilation, whereas the coincidental blockade of dopamine receptors in the corpus striatum is thought to cause muscular rigidity, which generates heat [69]. The combination of these processes is suggested to lead to the rare but serious adverse reaction to neuroleptics known as neuroleptic malignant syndrome, which is characterized by high fever and muscle rigidity.

Antidepressants, such as the dual dopamine/noradrenaline reuptake inhibitor bupropion, have been shown to significantly increase core temperature in exercising humans [70] and both core and brain temperature in exercising rats [71]. As bupropion in animal experiments has been shown to increase dopamine levels in the POAH [71], this effect contradicts studies discussed previously. However, several studies have questioned the dopaminergic efficiency of this drug in humans [72,73] and instead suggest that its clinical effect is mediated by the actions of noradrenaline [74] or nitric oxide (NO) signaling pathways [72], which may also influence its effect on body temperature.

Thirst perception may be disturbed by drugs at several levels of the regulation system [75], which contributes to the development of dehydration. Antipsychotic drugs, such as clozapine, risperdone, and olanzapine, have been reported to reduce thirst in human patients with polydipsia (excessive thirst); however, it is not known if these drugs have the same effect in non-polydipsic patients [75]. Through various pathways, thirst is also reduced in patients treated with drugs containing dopamine D1 and D2 receptor agonists and selective serotonin reuptake inhibitors (SSRIs) [75].

An increase in the number of cases of drug-induced hyponatremia (a condition of abnormally low blood sodium levels below 130 mEq/L) have been reported during the warm seasons in Sweden [76]. Consumption of the most widely prescribed antidepressants, SSRIs, is known to be associated with inappropriate antidiuretic hormone secretion (SIADH) [77,78,79], which is characterized by hyponatremia, either caused by overstimulation of the release of ADH, or by increased renal responsiveness to ADH [80,81]. The mechanism by which SSRIs cause hyponatremia remains unclear, but it has been suggested that, SSRIs, to some degree, affect noradrenaline reuptake. The resulting increase in noradrenaline levels could stimulate ADH release via α-adrenergic receptors and lead to SIADH induction [81]. Cases suggestive of hyponatremia secondary to SIADH have been reported with the use of fluoxetine, paroxetine, sertraline, fluvoxamine, and citalopram.

## 6. Heat and Brain

In addition to heat fatigue, heat cramps and other organ system dysfunction, heat exposure may cause CNS abnormalities. Several studies have provided evidence that heat-exposure impairs cognitive function, disturbs execution of effective behavioral responses, and decreases the capacity of both working and short-term memory [82]. It is possible, that heat-induced changes in neural mechanisms underlying these disturbances may also affect and exacerbate symptoms in mental health patients during heat waves. A few studies, reviewed in Section 6.2., have investigated heat-related disruption in brain physiology, activity, and functional connectivity by using different neuroimaging techniques. However, to facilitate the understanding of the studies reviewed below, the methodology used in these studies will be shortly introduced and described in Section 6.1.

### 6.1. Background: Neuroimaging and Brain Connectivity

One of the major challenges in neuroscience has been to connect the anatomy of the brain to its functions. Since the late 1990s, neuroimaging has become the predominant technique to study the functional architecture of the brain. Many different neuroimaging methods exist; however, functional magnetic resonance imaging (fMRI) and electroencephalogram (EEG) are the predominant methods used in relevant studies, and so these two are briefly described below.

Changes in oxygen usage and regional cerebral blood flow mirror the degree of activity of regional neurons both during task performance and in the resting state [83]. The fMRI technique uses the magnetic properties of oxygenated and deoxygenated hemoglobin to create images of changing blood flow in the brain associated with neural activity [84]. These signal changes are referred to as blood oxygenation level-dependent (BOLD) contrast [83,85]. Depending on the clinical or scientific question, fMRI images can be used to investigate differences between clinical groups or to relate brain responses to environmental or contextual changes. The main limitations of fMRI are that it detects neural activity indirectly through the associated hemodynamic variations, and the general noisiness of the signals [84].

The EEG is a mostly non-invasive electrophysiological monitoring method that records the spontaneous electrical activity of the brain over time. Through multiple electrodes placed on the scalp, the EEG measures voltage fluctuations resulting from ionic current within the firing neurons of the brain [86]. Compared to fMRI, EEG is a much cheaper and simpler method for brain activity measurement, and it is widely used to diagnose epilepsy and other brain disorders. The EEG is also a popular tool in neuroscience, cognitive science, cognitive psychology, neurolinguistics, and psychophysiological research. However, one of the big disadvantages of the EEG is that it is hard to determine the exact location in the brain from which the electrical activity originates. Combining the EEG-fMRI techniques has been suggested to be beneficial for obtaining the most informative results [84].

Generally, neuroimaging studies are designed to investigate one of the two aspects of brain functions: localization or connectivity. When investigating localization, the goal of the study is to identify a functionally specialized region of the brain that is activated in response to a specific input or behavior. However, as most of the brain’s functions are based on coordinated interactions of large numbers of neurons distributed across different brain areas, the second concept, connectivity, is of increasing interest for both psychiatrists and neuroscientists. Connectivity analyses investigate the way in which brain regions communicate with one another [87]. The methods that are used for studying brain connectivity largely depend on the type of connectivity of interest. Structural connectivity analyses typically concentrate on identifying fiber bundles connecting different regions of the brain by using diffusion magnetic resonance imaging (MRI). Structural connectivity often reflects the functional connectivity [88], which is defined as a solely statistical dependency among remote neurophysiological events. Functional connectivity is typically inferred by the temporal correlation between fMRI BOLD or EEG signals acquired from separate regions of the brain. Effective connectivity refers to causal dynamics between different brain nodes (the influence one node exert over another), observed by using either BOLD or EEG signals [89].

“Brain Network” is an important concept in exploratory neuroimaging studies. Brain Networks are typically defined as brain regions that show functional connectivity (statistically correlated BOLD or other signal fluctuations detected by fMRI or EEG) under specific conditions. Brain imaging studies often concentrate on detecting certain context- or environment-related changes in specific brain networks. Many different brain networks have been identified. For a more detailed review, the reader is referred to Wig 2017 [90].

Functional connectivity analyses are increasingly used to study brain connectivity patterns in both clinical and research contexts. Depending on the research goals, different methodological approaches are used. In many studies, the researchers use carefully designed behavioral tasks that the subjects perform during the brain scanning process. However, since a number of clinical populations are unable to perform tasks for a variety of reasons but can still be studied at rest, the resting state fMRI (rs-fMRI) has emerged as a valuable method for studying brain connectivity. In rs-fMRI studies, participants are not required to perform any motor or cognitive task or to pay attention to any particular stimulus, but are instead instructed to clear their minds and not to engage in specific thoughts or visual images [83]. Rs-fMRI functional connectivity analyses thus revolve around detecting temporally correlated BOLD signals from distinct regions of the brain in the absence of any cognitive task [83,87]. Alterations in resting-state functional connectivity were shown to be associated with a wide variety of brain and behavioral disorders, such as attention deficit hyperactivity disorder (ADHD) [91], Alzheimer’s disease [92], obsessive compulsive disorder [93], depression [94], bipolar disorder [95], and schizophrenia [96]. Furthermore, functional connectivity analyses have revealed a discoordination in task-related networks in patients with generalized anxiety disorder [97], social anxiety disorder in adolescents [98] and adults [99], as well as specific phobia [100], pointing to a dysregulation of the fronto-amygdalar interplay [101].

### 6.2. Altered Brain Function in Hot Conditions–Evidence from Neuroimaging Studies

In 2013, Liu et al. [102] studied the effects of passive heat exposure on the neural mechanisms of the human attention network. The human attention network is thought to be composed of three separate yet intimately interacting networks that control the alerting, orienting, and executive functions [103]. The alerting network maintains a state of high sensitivity during task performance over a short period of time. It engages the frontal and parietal brain regions and thalamus and is thought to be regulated by the norepinephrine system [102,104]. The orienting network entails selective adjustments that direct attention toward a particular modality, spatial location, or stimulus feature [103]. For example, the orienting network allows attention to be disengaged from one focus and to be re-engaged on a new pathway of interest. Activation of prefrontal and parietal areas of the brain, including the superior parietal cortex and temporal parietal junction, underlies the orienting function. The orienting network is believed to be regulated by the cholinergic (acetylcholine-transmitting) systems in the basal forebrain [102]. The executive control network is associated with monitoring for and resolving conflicting mental states. This network activates the frontal lobe, especially in the midline frontal areas, such as the anterior cingulate cortex (associated with monitoring), and the lateral prefrontal cortex (associated with inhibiting irrelevant responses). Dopamine binding in the anterior cingulate cortex is thought to drive the executive function [102].

In the study by Liu et al. [102], the function of all three networks comprising the attention network was investigated during cognitive performance and under either temperate (25 °C) or hot (50 °C) conditions. The performance results suggested that only executive function was impaired by the heat exposure, whereas orienting and alerting were unaffected. However, the fMRI analyses showed that all three of the networks were significantly affected in hot conditions.

The significant impairment of executive function during the performance test suggests that the loss of being able to handle cognitive conflicts may be the most noticeable behavioral change in hyperthermic conditions. The authors suggested that the physiological pathway possibly explaining this impairment is mediated by the body temperature-induced increase in plasma serotonin levels, which results in the inhibition of central dopamine production. As dopamine is believed to regulate the activation of the executive brain network, such inhibition may lead to impaired executive function. However, a the orienting and alerting functions were clearly affected by the heat exposure in the fMRI analysis but not in the performance tests, the authors concluded that a compensatory pathway involving other brain regions may become activated under stressful conditions, such as heat exposure.

Since neuronal synchrony and connectivity is impaired in several mental disorders, such compensatory activation of various brain regions may not be possible in this group of patients and may therefore increase their vulnerability during prolonged heatwaves. Furthermore, as the attention network, and especially the executive function, has been found to be deficient in several groups of mental health patients, such as sufferers of depression [105] and ADHD [106], heat exposure may further burden an already weakened function.

Shibasaki et al. [107] assessed the effects of heat stress on the cognitive processing of human motor control of executive and inhibitory responses. These responses were tested with a somatosensory Go/No-Go task, in which the participant received cutaneous “Go” and “No-go” signals and was asked to respond by pushing a button as quickly as possible, but only after presentation of the Go signal. By using EEG recordings, they demonstrated that the neural activities of both response execution and response inhibition were decreased during heat exposure. Whereas facial cooling quickly returned cerebral blood flow and the self-reported thermal comfort to baseline levels, the executive and inhibitory processing remained reduced for a longer period. Whole body cooling recovered executive processing, whereas inhibitory processing remained reduced. These results suggest that heat stress likely affects cognitive processing in the brain even after thermal comfort is regained.

Sun et al. [82] studied the effects of passive heat exposure on human brain functional connectivity patterns. Two different kinds of analysis methods, seed-based and node-based, were used to process the data from fMRI resting state functional connectivity analyses (rs-fMRI). The difference between these methods is as follows. In the seed-based correlation analyses, one region of interest (ROI) is defined in the brain, and a map, describing the strength of functional connectivity between the ROI and the rest of the brain regions, is obtained [87]. In the node-based analyses, the entire brain, or the entire explored area, is divided into “nodes”, i.e., regions that are considered to be functionally homogeneous, and the connectivity between the nodes is measured by pair-wise correlations.

The posterior cingulate cortex and precuneus (PCC/PCu) was defined as the ROI in the seed-based analyses by Sun et al. [64]. This area was chosen mainly for being a key metabolic area in the human default mode network (DMN), which is a resting state network consisting of regions that typically show slower activity during task performance than during the state of rest [88]. This area was also chosen, in part, because an abnormal PCC/PCu connectivity has been previously found to be associated with several neuropsychiatric disorders [91,92]. In the node-based connectivity analyses, the brain was divided into 90 separate regions and the correlation strength between each pair of these was estimated. Similar to the [102], the temperature in the heat-exposed group was kept constant at 50 °C and at 25 °C in the control group.

The results from the seed-based analysis showed a general trend toward significantly decreased connectivity between the PCC/PCu region and a number of brain areas when exposed to heat, such as the medial orbitofrontal cortex (mOFC) and medial temporal cortex [82]. mOFC is a region in the frontal lobes that is thought to be involved in self-evaluation and decision-making, including stimulus-reward associations and reinforcement of behavior [108,109]. One of the few increased connectivities in the seed-based analysis, detected between the PCC/PCu and the bilateral orbital superior frontal gyrus (associated with self-awareness), was suggested to be a compensatory reallocation of the mOFC cognitive resources [82]. Regions in the partial medial temporal lobe (especially the hippocampus and parahippocampal gyrus) are essential for normal human learning, memory, and spatial location. According to several earlier reports, both learning and memory can be impaired by heat-exposure in the performance tests [110,111]. The findings of Sun et al. regarding the heat-induced decrease in the connectivity between the ROI and the regions in partial medial temporal lobe thus provided neuroimaging evidence for previous cognition studies [82]. In addition, the within-region connectivity of the ROI itself was affected by heat, suggesting abnormalities in the function of this region during hyperthermia. This finding may explain some previous evidence from behavioral studies suggesting impaired self-referential cognitive ability during hyperthermia [112].

In the node-based analysis, 65 disturbed functional connectivities, 50 of which decreased (77%) and 15 increased, were found in the heat-exposed conditions compared with the temperate conditions [82]. Similar to the seed-based analysis, the most heat-affected region was the mOFC. In addition, heat exposure decreased the connectivity patterns in the temporal and occipital lobes. Increased connectivity in association with heat exposure was mainly located within the limbic system and was suggested to be related to the sensation of thermal discomfort and activation of thermoregulatory mechanisms [82]. Although the physiological and psychological consequences of the detected connectivity disturbances are still a matter of speculation, heat exposure clearly affects both inter- and intra-regional information processing and transmission, and thereby influences brain function and cognitive behavior through many different pathways [82].

The findings of Sun et al. are interesting from several points of view. Firstly, by demonstrating altered brain connectivity patterns associated with many cognitive functions in healthy individuals in hot environments, they provided neurological evidence for earlier findings from performance tests. Secondly, they provide a basis for speculation about the nature of vulnerability in mental health patients, as several of the psychiatric disorders entail disrupted brain connectivity without being exposed to heat. For example, decreased connectivity between PCC/PCu and mOFC, similar to what was reported by Sun et al., during heat-exposure in healthy individuals, has been previously observed in individuals with social anxiety disorder under unmanipulated temperature conditions [101]. Furthermore, lower connectivity between PCC/PCu and the medial temporal lobe, also observed by Sun et al., has been described in individuals with Alzheimer’s disease [92]. Adding the burden of high temperatures to already disrupted neurological functions may have a deleterious effect on the capacity to execute appropriate behavioral and cognitive responses, and may therefore influence the morbidity and mortality of mental health patients during heat waves.

Further evidence for heat-related changes in central neurological networks was reported by Qian et al. [113]. In this study, the influence of passive heat exposure (50 °C) on human brain functional network topological patterns was examined using a node-based method called the graph theory analysis. The ‘graph’ in this case expresses the network matrix of nodal connections. As the measures of this analysis method contained several specific terms that are most likely not recognized by non-specialists, we will describe the most common estimates bellow.

Minimum path length is a central characteristic in graph theory analysis that expresses the smallest number of nodal connectivity needed to travel from node A to B. The clustering coefficient of a node is a measure of the number of other nodes that are connected to it, which also are connected to each other. A high clustering coefficient means then that if nodes A and B are both connected to C, they are also likely to be connected to each other [87]. By using the local estimates of minimum path length and clustering coefficient, the mean path length and mean clustering coefficient can be calculated for the whole graph. Global efficiency, which is inversely proportional to the average minimum path length, is used to express the efficiency of the graph as a whole. Furthermore, small worldness in the brain implies that the nodes are densely connected locally, with a few long-distance connections. This kind of organizational pathway requires low minimum path lengths and has a high clustering coefficient. The small worldness of a network is estimated by comparing the small-world average minimum path length and average clustering coefficient with a randomly connected network [87].

Qian et al. [93] found that brain topological patterns differed between the control and heat-exposed groups. The clustering coefficients and small-worldness significantly decreased in the heat-exposed group, implying lower functional network connectivity. In addition, the network efficiency analyses revealed significantly decreased local and global efficiency in hypothermic conditions, entailing lower efficiency in transmitting information in both local and global level brain networks. Altogether, these alterations might result in brain functional disorders and cognitive performance decline [113]. Furthermore, the decrease in clustering coefficient, lower local efficiency, but maintained shortest path length suggest that functional networks during hyperthermia shift from an efficient small-worldness mode to a more randomized network with a less optimal topological organization. Disruptions in the topological organization and reduced small-worldness have also been associated with, for example, schizophrenic [114] and bipolar disorders [115].

Altered distribution in neural activity during heat stress was furthermore supported by a study investigating the effects of environmental heat on patterns of cerebral blood flow [116]. As discussed above, one of the central adaptive physiological responses to heat is peripheral vasodilation, resulting in markedly increased skin blood flow. However, this process was speculated to lead to a competition of blood supply between the periphery and the core (the latter including the brain), necessitating the prioritization of certain functions and brain regions by central mechanisms. Supporting this theory, several studies reported that the mean blood velocity in the cerebral artery decreases during heat exposure [107,117,118,119]. However, other (mostly older) studies have provided evidence that the cerebral circulatory system has an inherent ability to maintain cerebral blood flow (CBF) stable, and is therefore able to meet normal neural activity demands during environmental and physiological variation [120]. Qian et al. [116] quantified the resting state CBF under hot and temperate conditions (50 °C versus 25 °C) by using arterial spin labeling (ASL) MRI. ASL studies require that arterial blood water is first magnetically labeled (for example, by applying a radiofrequency inversion pulse) and then imaged by MR. After a 60 min exposure, Qian et al. [96] detected a non-significant tendency toward decreased general CBF in the heat-exposed group. In addition, they found significant regional blood redistribution in several brain regions, including the prefrontal cortex, somatosensory areas, and the limbic system in the heat-stressed individuals. Induced by increased body temperatures, the blood flow increased in the dorsal hypothalamus, posterior cingulate gyrus, and right middle cingulate, reflecting an increased regional blood demand for central thermoregulation activity, and a psychological sensation of discomfort in heat-exposed persons [116]. At the same time, decreased CBF in parts of the limbic system (such as, the parahippocampal gyrus, hippocampus, and amygdala) were mirrored by altered mood states, such as increased nervousness and anger detected during performance tests. This study provided evidence that the reorganization of neuronal activity in the brain during heat stress is most likely adaptive and based on prioritizing the most important task for the moment. However, if the heat exposure becomes more long-term, the resulting changes in cognitive and mood function may possibly increase the vulnerability of persons with already weakened mental health.

The above studies provided evidence for altered brain activity and connectivity during heat exposure. However, as the experimental procedures entail only acute effects caused by short periods of rather high temperature exposure, they are unlikely to fully capture the effects of long-lasting heat waves. As the brain dysfunctions caused by heat in many cases seem to be similar to those caused by different mental diseases, we can suspect that heat may, at certain cases, have an exacerbating effect on disease symptoms.

## 7. Heatwave Induced Sleep Disruptions—A Possible Pathway to Mental Health Problems

Human sleep is sensitive to environmental characteristics and even minor environmental changes may lead to sleep disturbances and sleep deprivation [121]. Surprisingly, the effects of heatwaves on sleep quality and the resulting health effects have not been frequently studied. A handful of hot chamber studies have demonstrated that high night temperatures lead to sleep disturbances in the form of reduced slow-wave (SWS) and rapid eye movement (REM) sleep [122,123,124], frequent sleep interruptions [125], and increased body movements, especially in the elderly [126,127]. Experimental data also show that heat exposure can lead to opposite effects, depending on whether it occurs several hours before or during sleep. While high air temperatures in the bedroom appear to only exert negative effects on sleep quality, baths and saunas before night rest have been shown to increase SWS [121,128]. Thus, a heatwave with greatly elevated night temperatures is probably more detrimental for sleep than high temperatures during daytime only. Acclimatization has been shown to improve human capacity to sleep under hot environmental conditions; however, in people that are not acclimatized, high nighttime temperatures are almost always deleterious to sleep quality [121]. Consequently, the potential health effects of sleep-deprivation are likely to increase during heatwaves.

Sleep deprivation strongly correlates with increased mortality and morbidity in epidemiological reports [129]. In experimental studies, the stress of sleep deprivation has been found to trigger increases of both pro- and anti-inflammatory proteins and blood pressure, alter output of metabolic hormones, and increase late-day levels of cortisol [129,130,131]. Many of these acute responses to sleep deprivation are similar to those seen in profiles predicting future risks of obesity, diabetes, cardiovascular diseases, and psychiatric disorders.

Clinical evidence suggest that sleep and emotion interact, and that nearly all psychiatric and neurological disorders (i.e., schizophrenia, affective disorders, addictions, dementia, and many more) are associated with sleeping problems [132,133]. Some evidence also suggested that sleeping disorders contribute to the formation of new mental health problems and to the maintenance of existing ones [133,134,135,136]. Baglioni et al., for example, conducted a meta-analysis of 21 studies investigating the longitudinal associations between sleep deprivation and depression, and concluded that non-depressed people with insomnia have a twofold risk of developing depression compared to people with no sleep difficulties [135]. Jansson-Fröjmark and Lindblom examined the directional associations between insomnia, anxiety, and depression and found that whereas insomnia increased the likelihood of developing anxiety and depression, the occurrence of these mental disorders also increases the probability of new onset insomnia [137]. Sleep is thereby thought to have a bidirectional relationship with mental health, and sleeping problems are believed to influence both the onset and the trajectory of mental disorders [133]. However, in terms of heatwave-induced sleep-deprivation, the periods involving heat-induced interrupted sleep are probably not sufficiently long to generate new cases of mental diseases; still, these are likely to contribute to the maintenance and exacerbation of already present mental health symptoms. Sleeplessness is thought to affect people’s ability to cope with daily life both through its effects on emotional health and on cognitive capacity.

Sleep deprivation has been, in both children and adults, associated with difficulties with focused attention and increased emotional reactivity, including low threshold, irritability, easy frustration, and difficulty modulating impulses [138,139,140]. Sleep loss also amplifies the negative emotions from disruptive events while reducing the positive effect of goal-enhancing events and elevating the baseline for positive emotions [141]. Changes in amygdalal activity in the brain have been suggested to be involved in sleep deprivation-related lack of emotional control [132]. The amygdala has a well-documented role in the processing of emotional information and, in particular, aversive stimuli. The extent of amygdala engagement in emotional processing is influenced by a variety of central systems, but particularly by the medial-prefrontal cortex (MPFC), which is thought to exert inhibitory control over amygdala function and contribute to contextually appropriate emotional responses [132].

Yoo et al. [132] used the fMRI technique to demonstrate that sleep deprivation inappropriately modulates the emotional brain response to negative aversive stimuli. During scanning, the two groups–one of which was previously exposed to approximately 35 h sleep deprivation and the other that slept normally–were asked to perform an emotional stimulus-viewing task, involving a presentation of 100 images depicting emotionally neutral to increasingly aversive stimuli. The authors compared amygdala reactivity between the two groups and reported that the sleep-deprived group exhibited a 60% greater magnitude of amygdala activation relative to control group when exposed to aversive stimuli. Amygdala activation was similar in both groups when presented with neutral pictures. The authors also found a significantly weaker connectivity between the amygdala and MPFC in the sleep-deprived group than in the group that had slept normally, suggesting a failure in top-down, prefrontal control. However, a significantly greater amygdala connectivity with the autonomic activating centers of brainstem, such as the locus coeruleus (involved in physiological responses to stress and panic) and midbrain, (associated with vision, hearing, motor control, and alertness) was found in the sleep-deprived group. These data suggested that sleep deprivation creates an amplified hyper-limbic response by the amygdala to negative emotional stimuli, whereas normal sleep may be necessary to reset the correct brain reactivity to emotional challenges [132].

Increased negativity may appear to be a relatively minor and temporary problem; however, in some circumstances, its results may be devastating. For example, Ballard et al. reported that greater nocturnal wakefulness, especially during early morning hours, significantly increased next day suicidal thoughts in patients with major depression and bipolar disorder. Furthermore, a large study involving more than 10,000 adolescents in Norway reported that young individuals with sleep problems were significantly more likely to engage in self-harm than those without sleep problems [142]. Sleep deprivation-related emotional negativity may also result in reduced motivation to take care of oneself, including taking necessary prescription medications. Thus, during heatwaves that involve prolonged periods of low quality sleep, the emotional negativity caused by sleeplessness may seriously affect the motivation and increase risk-related behaviors of individuals with mental disorders.

Sleep deprivation is well-known to impair learning and memory [132,143]. At the cellular level, sleep deprivation impairs hippocampal cellular excitability (the neuronal ability to generate an action potential), necessary for inducing synaptic potentiation (a long-lasting increase in signal transmission between two neurons). As the modification of synaptic potentiation is one of the major cellular mechanisms that underlies learning and memory, this sleep deprivation-induced decay in long-lasting synaptic plasticity can result in reduced capacity of these functions [143].

Mental and neurological disorders are almost always accompanied by sleep problems; however, the severity of the sleep problems appears to affect the extent of associated cognitive dysfunction. Sleep quality in individuals with Parkinson disease, for example, seems to be significantly correlated with cognition characteristics, such as attention and executive function [144]. Kanady et al. [145] demonstrated that whereas increased sleep time variability was associated with poorer working memory and verbal learning performances during inter-episode phase in individuals with bipolar disorders, sleep treatment improved these cognitive tasks. These findings further illustrate the importance of sleep in sufferers of mental health disorders and suggest how the lack of sleep during heat waves may hamper their ability to cope with everyday life.

## 8. Conclusions

The frequency and intensity of heatwaves has increased globally, resulting in higher numbers of morbidity and mortality cases. Both the number of deaths and emergency department visits for mental health emergencies have been associated with periods of high ambient temperatures. The maladaptive effects of certain psychiatric medicines, as well as the decreased capacity for behavioral adaptation in mental disease patients, can partly explain these associations during heatwaves. However, hot weather may also be a triggering factor for changes in central neurophysiological signaling, and cause disruptions in sleep patterns, which may potentially further increase the vulnerability of mental health patients. An increased exchange of information between specialists from different disciplines would considerably increase our understanding of this health burden.

## References

[1] Ebi K.L., Ogden N.H., Semenza J.C., Woodward A. (2017). Detecting and Attributing Health Burdens to Climate Change. Environ. Health Perspect..

[2] De’ Donato F.K., Leone M., Scortichini M., De Sario M., Katsouyanni K., Lanki T., Basagaña X., Ballester F., Åström C., Paldy A. (2015). Changes in the Effect of Heat on Mortality in the Last 20 Years in Nine European Cities. Results from the PHASE Project. Int. J. Environ. Res. Public Health.

[3] World Meteorological Organization (2014). Atlas of Mortality and Economic Losses from Weather, Climate and Water Extremes (1970–2012).

[4] Åström C. (2017). Health Effects of Heatwaves: Short and Long Term Predictions.

[5] Kenny G.P., Yardley J., Brown C., Sigal R.J., Jay O. (2010). Heat stress in older individuals and patients with common chronic diseases. Can. Med. Assoc. J..

[6] Quinn A., Tamerius J.D., Perzanowski M., Jacobson J.S., Goldstein I., Acosta L., Shaman J. (2014). Predicting indoor heat exposure risk during extreme heat events. Sci. Total Environ..

[7] Robine J.-M., Cheung S.L.K., Le Roy S., Van Oyen H., Griffiths C., Michel J.-P., Herrmann F.R. (2008). Death toll exceeded 70,000 in Europe during the summer of 2003. C. R. Biol..

[8] Bouchama A., Dehbi M., Mohamed G., Matthies F., Shoukri M., Menne B. (2007). Prognostic factors in heat wave–related deaths: A meta-analysis. Arch. Intern. Med..

[9] Bark N. (1998). Deaths of psychiatric patients during heat waves. Psychiatr. Serv..

[10] Semenza J.C., Rubin C.H., Falter K.H., Selanikio J.D., Flanders W.D., Howe H.L., Wilhelm J.L. (1996). Heat-related deaths during the July 1995 heat wave in Chicago. N. Engl. J. Med..

[11] Semenza J.C., McCullough J.E., Flanders W.D., McGeehin M.A., Lumpkin J.R. (1999). Excess hospital admissions during the July 1995 heat wave in Chicago. Am. J. Prev. Med..

[12] Fouillet A., Rey G., Laurent F., Pavillon G., Bellec S., Guihenneuc-Jouyaux C., Clavel J., Jougla E., Hémon D. (2006). Excess mortality related to the August 2003 heat wave in France. Int. Arch. Occup. Environ. Health.

[13] Vandentorren S., Bretin P., Zeghnoun A., Mandereau-Bruno L., Croisier A., Cochet C., Ribéron J., Siberan I., Declercq B., Ledrans M. (2006). August 2003 Heat Wave in France: Risk Factors for Death of Elderly People Living at Home. Eur. J. Public Health.

[14] Hansen A., Bi P., Nitschke M., Ryan P., Pisaniello D., Tucker G. (2008). The effect of heat waves on mental health in a temperate Australian city. Environ. Health Perspect..

[15] Wang X., Lavigne E., Ouellette-kuntz H., Chen B.E. (2014). Acute impacts of extreme temperature exposure on emergency room admissions related to mental and behavior disorders in Toronto, Canada. J. Affect. Disord..

[16] Sung T.-I., Chen M.-J., Lin C.-Y., Lung S.-C., Su H.-J. (2011). Relationship between mean daily ambient temperature range and hospital admissions for schizophrenia: Results from a national cohort of psychiatric inpatients. Sci. Total Environ..

[17] Zhao D., Zhang X., Xie M., Cheng J., Zhang H., Wang S., Li K., Yang H., Wen L., Wang X. (2016). Is greater temperature change within a day associated with increased emergency admissions for schizophrenia?. Sci. Total Environ..

[18] Trang P.M., Rocklöv J., Giang K.B., Van Minh H., Tinh L.T., Nilsson M. (2015). Weather Variations and Hospital Admissions for Depressive Disorders: A Case Study in Hanoi. Ann. Psychiatry Ment. Health.

[19] Conti S., Masocco M., Meli P., Minelli G., Palummeri E., Solimini R., Toccaceli V., Vichi M. (2007). General and specific mortality among the elderly during the 2003 heat wave in Genoa (Italy). Environ. Res..

[20] Linares C., Culqui D., Carmona R., Ortiz C., Díaz J. (2017). Short-term association between environmental factors and hospital admissions due to dementia in Madrid. Environ. Res..

[21] Medici C.R., Vestergaard C.H., Hadzi-Pavlovic D., Munk-Jørgensen P., Parker G. (2016). Seasonal variations in hospital admissions for mania: Examining for associations with weather variables over time. J. Affect. Disord..

[22] Linares C., Martinez-Martin P., Rodríguez-Blázquez C., Forjaz M.J., Carmona R., Díaz J. (2016). Effect of heat waves on morbidity and mortality due to Parkinson’s disease in Madrid: A time-series analysis. Environ. Int..

[23] Culqui D., Linares C., Ortiz C., Carmona R., Díaz J. (2017). Association between environmental factors and emergency hospital admissions due to Alzheimer’s disease in Madrid. Sci. Total Environ..

[24] Cervellin G., Comelli I., Lippi G., Comelli D., Rastelli G., Ossola P., Marchesi C. (2014). The number of emergency department visits for psychiatric emergencies is strongly associated with mean temperature and humidity variations. Results of a nine year survey. Emerg. Care J..

[25] Trang P.M., Rocklöv J., Giang K.B., Kullgren G., Nilsson M. (2016). Heatwaves and Hospital Admissions for Mental Disorders in Northern Vietnam. PLoS ONE.

[26] Trang P.M., Rocklöv J., Giang K.B., Nilsson M. (2016). Seasonality of hospital admissions for mental disorders in Hanoi, Vietnam. Glob. Health Action.

[27] Morrison S.F., Nakamura K. (2011). Central neural pathways for thermoregulation. Front. Biosci..

[28] Smith C.J., Johnson J.M. (2016). Responses to hyperthermia. Optimizing heat dissipation by convection and evaporation: Neural control of skin blood flow and sweating in humans. Auton. Neurosci..

[29] Martin-Latry K., Goumy M.-P., Latry P., Gabinski C., Bégaud B., Faure I., Verdoux H. (2007). Psychotropic drugs use and risk of heat-related hospitalisation. Eur. Psychiatry.

[30] Yablonskiy D.A., Ackerman J.J., Raichle M.E. (2000). Coupling between changes in human brain temperature and oxidative metabolism during prolonged visual stimulation. Proc. Natl. Acad. Sci. USA.

[31] Sharma H.S., Hoopes P. (2003). Hyperthermia induced pathophysiology of the central nervous system. Int. J. Hyperth..

[32] Morrison S.F. (2016). Central neural control of thermoregulation and brown adipose tissue. Auton. Neurosci..

[33] Antunes-Rodrigues J., De Castro M., Elias L.L., Valença M.M., McCann S.M. (2004). Neuroendocrine control of body fluid metabolism. Physiol. Rev..

[34] Kellogg D., Hodges G.J., Orozco C.R., Phillips T.M., Zhao J.L., Johnson J.M. (2007). Cholinergic mechanisms of cutaneous active vasodilation during heat stress in cystic fibrosis. J. Appl. Physiol..

[35] Koch C.A., Fulop T. (2017). Clinical aspects of changes in water and sodium homeostasis in the elderly. Rev. Endocr. Metab. Disord..

[36] Millard-Stafford M., Wendland D.M., O’Dea N.K., Norman T.L. (2012). Thirst and hydration status in everyday life. Nutr. Rev..

[37] McLane V.D. (2011). Characterization of Thermoregulatory Efferents to the Paraventricular Nucleus of the Rat Hypothalamus.

[38] Chauhan N.R., Kapoor M., Singh L.P., Gupta R.K., Meena R.C., Tulsawani R., Nanda S., Singh S.B. (2017). Heat stress-induced neuroinflammation and aberration in monoamine levels in hypothalamus are associated with temperature dysregulation. Neuroscience.

[39] McMorris T., Swain J., Smith M., Corbett J., Delves S., Sale C., Harris R.C., Potter J. (2006). Heat stress, plasma concentrations of adrenaline, noradrenaline, 5-hydroxytryptamine and cortisol, mood state and cognitive performance. Int. J. Psychophysiol..

[40] Lustig R. (2017). The pursuit of pleasure is a modern-day addiction. The Guardian.

[41] Bell V. (2013). The unsexy truth about dopamine. The Guardian.

[42] Oaklander M. (2017). New Hope for Depression. Time.

[43] Lee C.-P., Chen P.-J., Chang C.-M. (2015). Heat stroke during treatment with olanzapine, trihexyphenidyl, and trazodone in a patient with schizophrenia. Acta Neuropsychiatr..

[44] Feldberg W., Myers R. (1963). A new concept of temperature regulation by amines in the hypothalamus. Nature.

[45] Ishiwata T., Hasegawa H., Greenwood B.N. (2017). Involvement of serotonin in the ventral tegmental area in thermoregulation of freely moving rats. Neurosci. Lett..

[46] Zheng X., Hasegawa H. (2016). Central dopaminergic neurotransmission plays an important role in thermoregulation and performance during endurance exercise. Eur. J. Sport Sci..

[47] Cox B., Davis A., Juxon V., Lee T., Martin D. (1983). A role for an indoleamine other than 5-hydroxytryptamine in the hypothalamic thermoregulatory pathways of the rat. J. Physiol..

[48] Ishiwata T., Saito T., Hasegawa H., Yazawa T., Otokawa M., Aihara Y. (2004). Changes of body temperature and extracellular serotonin level in the preoptic area and anterior hypothalamus after thermal or serotonergic pharmacological stimulation of freely moving rats. Life Sci..

[49] Zhang Y.H., Hosono T., Yanase-Fujiwara M., Chen X.M., Kanosue K. (1997). Effect of midbrain stimulations on thermoregulatory vasomotor responses in rats. J. Physiol..

[50] Ootsuka Y., Tanaka M. (2015). Control of cutaneous blood flow by central nervous system. Temperature.

[51] Björklund A., Dunnett S.B. (2007). Dopamine neuron systems in the brain: An update. Trends Neurosci..

[52] De Roij T.A., Frens J., Bakker J., Németh F. (1977). Thermoregulatory effects of intraventricularly injected dopamine in the goat. Eur. J. Pharmacol..

[53] Lee T., Mora F., Myers R. (1985). Dopamine and thermoregulation: An evaluation with special reference to dopaminergic pathways. Neurosci. Biobehav. Rev..

[54] Scott I., Boulant J.A. (1984). Dopamine effects on thermosensitive neurons in hypothalamic tissue slices. Brain Res..

[55] Brown S., Gisolfi C., Mora F. (1982). Temperature regulation and dopaminergic systems in the brain: Does the substantia nigra play a role?. Brain Res..

[56] Balthazar C.H., Leite L.H., Ribeiro R.M., Soares D.D., Coimbra C.C. (2010). Effects of blockade of central dopamine D 1 and D 2 receptors on thermoregulation, metabolic rate and running performance. Pharmacol. Rep..

[57] Robertson S.D., Plummer N.W., de Marchena J., Jensen P. (2013). Developmental origins of central norepinephrine neuron diversity. Nat. Neurosci..

[58] Clark W.G., Lipton J. (1986). Changes in body temperature after administration of adrenergic and serotonergic agents and related drugs including antidepressants: II. Neurosci. Biobehav. Rev..

[59] Watanabe T., Morimoto A., Murakami N. (1986). Effect of amine on temperature-responsive neuron in slice preparation of rat brain stem. Am. J. Physiol. Regul. Integr. Comp. Physiol..

[60] Meeusen R., Roelands B. (2010). Central fatigue and neurotransmitters, can thermoregulation be manipulated?. Scand. J. Med. Sci. Sports.

[61] Quan N., Xin L., Blatteis C.M. (1991). Microdialysis of norepinephrine into preoptic area of guinea pigs: Characteristics of hypothermic effect. Am. J. Physiol. Regul. Integr. Comp. Physiol..

[62] Quan N., Xin L., Ungar A., Blatteis C. (1992). Preoptic norepinephrine-induced hypothermia is mediated by alpha 2-adrenoceptors. Am. J. Physiol. Regul. Integr. Comp. Physiol..

[63] Batscha C.L. (1997). Heat stroke: Keeping your clients cool in the summer. J. Psychosoc. Nurs. Ment. Health Serv..

[64] Lieberman J.A. (2004). Managing Anticholinergic Side Effects. Prim. Care Companion J. Clin. Psychiatry.

[65] Kwok J.S., Chan T.Y. (2005). Recurrent heat-related illnesses during antipsychotic treatment. Ann. Pharmacother..

[66] Hayes B.D., Martinez J.P., Barrueto F. (2013). Drug-induced hyperthermic syndromes: Part I. Hyperthermia in overdose. Emerg. Med. Clin..

[67] Martinez M., Devenport L., Saussy J., Martinez J. (2002). Drug-associated heat stroke. South. Med. J..

[68] Kilbourne E.M., Choi K., Jones T.S., Thacker S.B. (1982). Risk factors for heatstroke: A case-control study. JAMA.

[69] Adnet P., Lestavel P., Krivosic-Horber R. (2000). Neuroleptic malignant syndrome. Br. J. Anaesth..

[70] Roelands B., Hasegawa H., Watson P., Piacentini M.F., Buyse L., De Schutter G., Meeusen R. (2009). Performance and thermoregulatory effects of chronic bupropion administration in the heat. Eur. J. Appl. Physiol..

[71] Hasegawa H., Meeusen R., Sarre S., Diltoer M., Piacentini M.F., Michotte Y. (2005). Acute dopamine/norepinephrine reuptake inhibition increases brain and core temperature in rats. J. Appl. Physiol..

[72] Dhir A., Kulkarni S. (2007). Involvement of nitric oxide (NO) signaling pathway in the antidepressant action of bupropion, a dopamine reuptake inhibitor. Eur. J. Pharmacol..

[73] Meyer J.H., Goulding V.S., Wilson A.A., Hussey D., Christensen B.K., Houle S. (2002). Bupropion occupancy of the dopamine transporter is low during clinical treatment. Psychopharmacology.

[74] Cooper B.R., Wang C.M., Cox R.F., Norton R., Shea V., Ferris R.M. (1994). Evidence that the Acute Behavioral and Electrophysiological Effects of Bupropion (Wellbutrin^®^) Are Mediated by a Noradrenergic Mechanism. Neuropsychopharmacology.

[75] Stöllberger C., Lutz W., Finsterer J. (2009). Heat-related side-effects of neurological and non-neurological medication may increase heatwave fatalities. Eur. J. Neurol..

[76] Jönsson A.K., Lövborg H., Lohr W., Ekman B., Rocklöv J. (2017). Increased Risk of Drug-Induced Hyponatremia during High Temperatures. Int. J. Environ. Res. Public Health.

[77] Gandhi S., Shariff S.Z., Al-Jaishi A., Reiss J.P., Mamdani M.M., Hackam D.G., Li L., McArthur E., Weir M.A., Garg A.X. (2017). Second-generation antidepressants and hyponatremia risk: A population-based cohort study of older adults. Am. J. Kidney Dis..

[78] Wilkinson T.J., Begg E.J., Winter A.C., Sainsbury R. (1999). Incidence and risk factors for hyponatraemia following treatment with fluoxetine or paroxetine in elderly people. Br. J. Clin Pharmacol..

[79] Shakibaei F., Gholamrezaei A., Alikhani M., Talaeizadeh K. (2010). Serum sodium changes in fluoxetine users at different age groups. Iran. J. Psychiatry.

[80] Kirpekar V.C., Joshi P.P. (2005). Syndrome of inappropriate ADH secretion (SIADH) associated with citalopram use. Indian J. Psychiatry.

[81] Jacob S., Spinier S.A. (2006). Hyponatremia Associated with Selective Serotonin-Reuptake Inhibitors in Older Adults. Ann. Pharmacother..

[82] Sun G., Qian S., Jiang Q., Liu K., Li B., Li M., Zhao L., Zhou Z., von Deneen K.M., Liu Y. (2013). Hyperthermia-induced disruption of functional connectivity in the human brain network. PLoS ONE.

[83] Takamura T., Hanakawa T. (2017). Clinical utility of resting-state functional connectivity magnetic resonance imaging for mood and cognitive disorders. J. Neural Transm..

[84] Vitali P., Di Perri C., Vaudano A.E., Meletti S., Villani F. (2015). Integration of multimodal neuroimaging methods: A rationale for clinical applications of simultaneous EEG-fMRI. Funct. Neurol..

[85] Nair V.A., Raut R.V., Prabhakaran V. (2017). Investigating the Blood Oxygenation Level-Dependent Functional MRI Response to a Verbal Fluency Task in Early Stroke before and after Hemodynamic Scaling. Front. Neurol..

[86] Junghöfer M., Peyk P., Flaisch T., Schupp H.T., Anders S., Ende G., Junghofer M., Kissler J., Wildgruber D. (2006). Neuroimaging methods in affective neuroscience: Selected methodological issues. Progress in Brain Research.

[87] Bijsterbosch J., Smith S., Beckmann C. (2017). Resting STate fMRI Functional Connectivity.

[88] Greicius M.D., Supekar K., Menon V., Dougherty R.F. (2009). Resting-state functional connectivity reflects structural connectivity in the default mode network. Cereb. Cortex.

[89] Park H.-J., Friston K. (2013). Structural and functional brain networks: From connections to cognition. Science.

[90] Wig G.S. (2017). Segregated Systems of Human Brain Networks. Trends Cogn. Sci..

[91] Castellanos F.X., Margulies D.S., Kelly C., Uddin L.Q., Ghaffari M., Kirsch A., Shaw D., Shehzad Z., Di Martino A., Biswal B. (2008). Cingulate-precuneus interactions: A new locus of dysfunction in adult attention-deficit/hyperactivity disorder. Biol. Psychiatry.

[92] Greicius M.D., Srivastava G., Reiss A.L., Menon V. (2004). Default-mode network activity distinguishes Alzheimer’s disease from healthy aging: Evidence from functional MRI. Proc. Natl. Acad. Sci. USA.

[93] Harrison B.J., Soriano-Mas C., Pujol J., Ortiz H., López-Solà M., Hernández-Ribas R., Deus J., Alonso P., Yücel M., Pantelis C. (2009). Altered corticostriatal functional connectivity in obsessive-compulsive disorder. Arch. Gen. Psychiatry.

[94] Anand A., Li Y., Wang Y., Wu J., Gao S., Bukhari L., Mathews V.P., Kalnin A., Lowe M.J. (2005). Activity and connectivity of brain mood regulating circuit in depression: A functional magnetic resonance study. Biol. Psychiatry.

[95] Wang F., Kalmar J.H., He Y., Jackowski M., Chepenik L.G., Edmiston E.E., Tie K., Gong G., Shah M.P., Jones M. (2009). Functional and structural connectivity between the perigenual anterior cingulate and amygdala in bipolar disorder. Biol. Psychiatry.

[96] Salvador R., Sarró S., Gomar J.J., Ortiz-Gil J., Vila F., Capdevila A., Bullmore E., McKenna P.J., Pomarol-Clotet E. (2010). Overall brain connectivity maps show cortico-subcortical abnormalities in schizophrenia. Hum. Brain Mapp..

[97] Etkin A., Prater K.E., Schatzberg A.F., Menon V., Greicius M.D. (2009). Disrupted amygdalar subregion functional connectivity and evidence of a compensatory network in generalized anxiety disorder. Arch. Gen. Psychiatry.

[98] Guyer A.E., Lau J.Y., McClure-Tone E.B., Parrish J., Shiffrin N.D., Reynolds R.C., Chen G., Blair R., Leibenluft E., Fox N.A. (2008). Amygdala and ventrolateral prefrontal cortex function during anticipated peer evaluation in pediatric social anxiety. Arch. Gen. Psychiatry.

[99] Zhao X.-H., Wang P.-J., Li C.-B., Hu Z.-H., Xi Q., Wu W.-Y., Tang X.-W. (2007). Altered default mode network activity in patient with anxiety disorders: An fMRI study. Eur. J. Radiol..

[100] Åhs F., Pissiota A., Michelgård Å., Frans Ö., Furmark T., Appel L., Fredrikson M. (2009). Disentangling the web of fear: Amygdala reactivity and functional connectivity in spider and snake phobia. Psychiatry Res..

[101] Hahn A., Stein P., Windischberger C., Weissenbacher A., Spindelegger C., Moser E., Kasper S., Lanzenberger R. (2011). Reduced resting-state functional connectivity between amygdala and orbitofrontal cortex in social anxiety disorder. NeuroImage.

[102] Liu K., Sun G., Li B., Jiang Q., Yang X., Li M., Li L., Qian S., Zhao L., Zhou Z. (2013). The impact of passive hyperthermia on human attention networks: An fMRI study. Behav. Brain Res..

[103] Klein R.M., Hassan T., Wilson G., Ishigami Y., Mulle J. (2017). The AttentionTrip: A game-like tool for measuring the networks of attention. J. Neurosci. Methods.

[104] Petersen S.E., Posner M.I. (2012). The Attention System of the Human Brain: 20 Years After. Annu. Rev. Neurosci..

[105] Paelecke-Habermann Y., Pohl J., Leplow B. (2005). Attention and executive functions in remitted major depression patients. J. Affect. Disord..

[106] Makris N., Biederman J., Valera E.M., Bush G., Kaiser J., Kennedy D.N., Caviness V.S., Faraone S.V., Seidman L.J. (2006). Cortical thinning of the attention and executive function networks in adults with attention-deficit/hyperactivity disorder. Cereb. Cortex.

[107] Shibasaki M., Namba M., Oshiro M., Kakigi R., Nakata H. (2017). Suppression of cognitive function in hyperthermia; From the viewpoint of executive and inhibitive cognitive processing. Sci. Rep..

[108] Walton M.E., Behrens T.E.J., Buckley M.J., Rudebeck P.H., Rushworth M.F.S. (2010). Separable Learning Systems in the Macaque Brain and the Role of Orbitofrontal Cortex in Contingent Learning. Neuron.

[109] Beer J.S., Lombardo M.V., Bhanji J.P. (2010). Roles of Medial Prefrontal Cortex and Orbitofrontal Cortex in Self-evaluation. J. Cogn. Neurosci..

[110] Gaoua N., Racinais S., Grantham J., El Massioui F. (2011). Alterations in cognitive performance during passive hyperthermia are task dependent. Int. J. Hyperth..

[111] Racinais S., Gaoua N., Grantham J. (2008). Hyperthermia impairs short-term memory and peripheral motor drive transmission. J. Physiol..

[112] Raichle M.E., MacLeod A.M., Snyder A.Z., Powers W.J., Gusnard D.A., Shulman G.L. (2001). A default mode of brain function. Proc. Natl. Acad. Sci. USA.

[113] Qian S., Sun G., Jiang Q., Liu K., Li B., Li M., Yang X., Yang Z., Zhao L. (2013). Altered topological patterns of large-scale brain functional networks during passive hyperthermia. Brain Cogn..

[114] Lynall M.-E., Bassett D.S., Kerwin R., McKenna P.J., Kitzbichler M., Muller U., Bullmore E. (2010). Functional Connectivity and Brain Networks in Schizophrenia. J. Neurosci..

[115] Kim D.-J., Bolbecker A.R., Howell J., Rass O., Sporns O., Hetrick W.P., Breier A., O’Donnell B.F. (2013). Disturbed resting state EEG synchronization in bipolar disorder: A graph-theoretic analysis. NeuroImage.

[116] Qian S., Jiang Q., Liu K., Li B., Li M., Li L., Yang X., Yang Z., Sun G. (2014). Effects of short-term environmental hyperthermia on patterns of cerebral blood flow. Physiol. Behav..

[117] Wilson T.E., Cui J., Zhang R., Crandall C.G. (2006). Heat stress reduces cerebral blood velocity and markedly impairs orthostatic tolerance in humans. Am. J. Physiol. Regul. Integr. Comp. Physiol..

[118] Ross E.Z., Cotter J.D., Wilson L., Fan J.-L., Lucas S.J., Ainslie P.N. (2012). Cerebrovascular and corticomotor function during progressive passive hyperthermia in humans. J. Appl. Physiol..

[119] Nelson M.D., Haykowsky M.J., Stickland M.K., Altamirano-Diaz L.A., Willie C.K., Smith K.J., Petersen S.R., Ainslie P.N. (2011). Reductions in cerebral blood flow during passive heat stress in humans: Partitioning the mechanisms. J. Physiol..

[120] Van Beek A.H., Claassen J.A., Rikkert M.G.O., Jansen R.W. (2008). Cerebral autoregulation: An overview of current concepts and methodology with special focus on the elderly. J. Cereb. Blood Flow Metab..

[121] Buguet A. (2007). Sleep under extreme environments: Effects of heat and cold exposure, altitude, hyperbaric pressure and microgravity in space. J. Neurol. Sc..

[122] Kendel K., Schmidt-Kessen W. (1973). The influence of room temperature on night-sleep in man (polygraphic night-sleep recordings in the climatic chamber). Sleep 1972.

[123] Buguet A., Allin L., Dittmar A., Muzet A., Peyrin L., Roussel B. (1983). Human reactions to chronic heat. Proc. Int. Union Physiol. Sci..

[124] Haskell E., Palca J., Walker J., Berger R., Heller H. (1981). Metabolism and thermoregulation during stages of sleep in humans exposed to heat and cold. J. Appl. Physiol..

[125] Henane R., Buguet A., Roussel B., Bittel J. (1977). Variations in evaporation and body temperatures during sleep in man. J. Appl. Physiol..

[126] Ohnaka T., Tochihara Y., Kanda K. (1995). Body movements of the elderly during sleep and thermal conditions in bedrooms in summer. Appl. Hum. Sci..

[127] Okamoto-Mizuno K., Mizuno K. (2012). Effects of thermal environment on sleep and circadian rhythm. J. Physiol. Anthropol..

[128] Libert J.-P., Bach V. (2005). Thermoregulation and sleep in the human. The Physiologic Nature of Sleep.

[129] Mullington J.M., Haack M., Toth M., Serrador J.M., Meier-Ewert H.K. (2009). Cardiovascular, Inflammatory, and Metabolic Consequences of Sleep Deprivation. Prog. Cardiovasc. Dis..

[130] Frey D.J., Fleshner M., Wright K.P. (2007). The effects of 40 hours of total sleep deprivation on inflammatory markers in healthy young adults. Brain Behav. Immun..

[131] Krysta K., Krzystanek M., Bratek A., Krupka-Matuszczyk I. (2017). Sleep and inflammatory markers in different psychiatric disorders. J. Neural Transm..

[132] Yoo S.-S., Gujar N., Hu P., Jolesz F.A., Walker M.P. (2007). The human emotional brain without sleep—A prefrontal amygdala disconnect. Curr. Biol..

[133] Scott A.J., Webb T.L., Rowse G. (2017). Does improving sleep lead to better mental health? A protocol for a meta-analytic review of randomised controlled trials. BMJ Open.

[134] Freeman D., Stahl D., McManus S., Meltzer H., Brugha T., Wiles N., Bebbington P. (2012). Insomnia, worry, anxiety and depression as predictors of the occurrence and persistence of paranoid thinking. Soc. Psychiatry Psychiatr. Epidemiol..

[135] Baglioni C., Battagliese G., Feige B., Spiegelhalder K., Nissen C., Voderholzer U., Lombardo C., Riemann D. (2011). Insomnia as a predictor of depression: A meta-analytic evaluation of longitudinal epidemiological studies. J. Affect. Disord..

[136] Baglioni C., Nanovska S., Regen W., Spiegelhalder K., Feige B., Nissen C., Reynolds C.F., Riemann D. (2016). Sleep and mental disorders: A meta-analysis of polysomnographic research. Psychol. Bull..

[137] Jansson-Fröjmark M., Lindblom K. (2008). A bidirectional relationship between anxiety and depression, and insomnia? A prospective study in the general population. J. Psychosom. Res..

[138] Baglioni C., Spiegelhalder K., Lombardo C., Riemann D. (2010). Sleep and emotions: A focus on insomnia. Sleep Med. Rev..

[139] Dahl R.E. (1996). The impact of inadequate sleep on children’s daytime cognitive function. Seminars in Pediatric Neurology.

[140] Franzen P.L., Siegle G.J., Buysse D.J. (2008). Relationships between affect, vigilance, and sleepiness following sleep deprivation. J. Sleep Res..

[141] Zohar D., Tzischinsky O., Epstein R., Lavie P. (2005). The effects of sleep loss on medical residents’ emotional reactions to work events: A cognitive-energy model. Sleep.

[142] Hysing M., Sivertsen B., Stormark K.M., O’connor R.C. (2015). Sleep problems and self-harm in adolescence. Br. J. Psychiatry.

[143] Abel T., Havekes R., Saletin J.M., Walker M.P. (2013). Sleep, Plasticity and Memory from Molecules to Whole-Brain Networks. Curr. Biol..

[144] Stavitsky K., Neargarder S., Bogdanova Y., McNamara P., Cronin-Golomb A. (2012). The impact of sleep quality on cognitive functioning in Parkinson’s disease. J. Int. Neuropsychol. Soc..

[145] Kanady J.C., Soehner A.M., Klein A.B., Harvey A.G. (2017). The association between insomnia-related sleep disruptions and cognitive dysfunction during the inter-episode phase of bipolar disorder. J. Psychiatr. Res..

